# Effect of a Short, Animated Storytelling Video on Transphobia Among US Parents: Randomized Controlled Trial

**DOI:** 10.2196/66496

**Published:** 2025-01-20

**Authors:** Doron Amsalem, Merlin Greuel, Shuyan Liu, Andrés Martin, Maya Adam

**Affiliations:** 1 Department of Psychiatry, Columbia University, New York, NY, United States; 2 University Hospital, Heidelberg Institute of Global Health, Heidelberg University, Heidelberg, Germany; 3 Department of Psychiatry and Psychotherapy, Charité—Universitätsmedizin, Campus Charité Mitte, Berlin, Germany; 4 German Center for Mental Health, Deutsche Zentrum für Psychische Gesundheit, Berlin, Germany; 5 Child Study Center, Yale School of Medicine, Yale University, New Haven, CT, United States; 6 Faculty of Medicine, Tel Aviv University, Tel Aviv, Israel; 7 Stanford Department of Pediatrics, Stanford University School of Medicine, Stanford University, 453 Quarry Rd, Palo Alto, CA, 94304, United States, 1 6508393600; 8 Stanford Center for Digital Health, Stanford University School of Medicine, Stanford University, Stanford, CA, United States

**Keywords:** public health communication, vulnerable population, stigma reduction, stigma, transphobia, transgender, gender diverse, LGBTQ, parent, mental health, mental illness, transgender children, children, youth, adolescent, storytelling, animation

## Abstract

**Background:**

Parents play a pivotal role in supporting transgender and gender diverse (TGD) youth. Yet only 35% of TGD youth describe their home as a gender-affirming place. Lack of parental support contributes to recent findings that TGD youth are approximately three times more likely to attempt suicide than their cisgender peers. In contrast, parents’ affirmation of their children’s gender identity significantly improves their mental health outcomes, by reducing anxiety, depression, and suicidality.

**Objective:**

Addressing the urgent need for effective, scalable interventions, this study evaluates a novel digital approach: short, animated storytelling videos. We hypothesized that our 2.5-minute video intervention would reduce antitransgender stigma, or transphobia, and improve attitudes toward gender diverse children among US parents.

**Methods:**

We recruited 1267 US parents, through the Prolific Academic (Prolific) online research platform, and randomized them into video intervention or control groups. We measured transphobia using the Transgender Stigma Scale, and attitudes toward transgender children using the gender thermometer*,* before and after watching the video. We compared outcomes between the two groups using 2 × 3 ANOVA. Both groups were invited to return 30 days later for follow-up assessment, before being offered posttrial access to the intervention video, which portrayed an authentic conversation between a mother and her transgender child.

**Results:**

Single exposure to a short, animated story video significantly reduced transphobia and improved attitudes toward transgender children among US parents, immediately post intervention. We observed a significant group-by-time interaction in mean Transgender Stigma Scale scores (*F*_2,1_=3.7, *P*=.02) and significant between-group changes when comparing the video and control groups from baseline to post intervention (*F*_1_=27.4, *P*<.001). Effect sizes (Cohen *d*) indicated small to moderate immediate changes in response to the 2.5-minute video, though the effect was no longer observed at the 30-day follow-up. Gender thermometer scores revealed significant immediate improvements in the attitudes of participants in the video intervention arm, and this improvement was sustained at the 30-day time point.

**Conclusions:**

Short, animated storytelling is a novel digital approach with the potential to boost support and affirmation of transgender children, by offering authentic insights into the lived experiences of TGD youth. Repeated exposures to such interventions may be necessary to sustain improvements over time. Future studies could test a series of short, animated storytelling videos featuring the lived experiences of several TGD youth. Evaluating the effect of such a series could contribute to the fields of digital health communication and transgender health. Digital approaches, such as short, animated storytelling videos, that support empathy and acceptance of TGD youth could foster a more inclusive society in which every child can thrive.

## Introduction

Optimal health outcomes for transgender and gender diverse (TGD) youth depend on the support and affirmation of their parents, as well as other adults with whom they interact [1-5]. Yet, even in their own homes, only 35% of TGD youth feel they are in a gender-affirming place [6]. Lack of parental support contributes to recent findings that TGD youth are 2.7‐3.5 times more likely to attempt suicide than their cisgender peers [7,8]. Simple, gender-affirming acts, such as consistently respecting a child’s pronouns in the home, can dramatically reduce the percent of TGD youth who attempt suicide from 21% to 12% as in a 2023 US national survey [6]. However, reducing stigma toward TGD youth, among adults, remains challenging [9,10]. Stigma acts as a broader social mechanism that contributes to transphobia (prejudice against individuals who defy traditional gender norms) [9,11]. Recent findings indicate that, even within their own homes, only 35% of TGD youth report feeling that they are in a gender-affirming place, with parental transphobia being a key contributing factor [6]. Lack of adult support and affirmation remains one of the leading causes of poor mental health outcomes in TGD youth [2-5,12,13].

This public health problem is aggravated by a growing climate of general societal intolerance in the United States. A record number of anti-LGBTQ (lesbian, gay, bisexual, transgender, queer) bills (more than 400) were introduced across US state legislatures in 2023 [14]. These environmental factors contribute to an alarmingly high rate of suicidality among TGD youth: the 2023 US National Survey on the Mental Health of LGBTQ+ Young People reported that 48% of transgender girls, 56% of transgender boys, and 48% of nonbinary youth had seriously considered attempting suicide in the past year [6]. Overall, TGD youth are 3.5 times more likely to attempt suicide than their cisgender peers [8]. Simple gender-affirming acts from adults, such as consistently respecting a child’s pronouns, have been associated with a dramatic reduction in the percent of TGD youth who attempt suicide from 21% to 12% [6]. Even before the COVID-19 pandemic, a scoping review published in the *Journal of Adolescent Health* identified an urgent need for innovative interventions to reduce transphobia toward TGD youth [15]. Pandemic lockdowns, through a loss of community and school support, further threatened the mental health of TGD youth, who found themselves isolated and unsupported [16-18].

Research on transphobia reduction in schools highlights the need for interventions to reduce stigma among all parents, not just those of TGD youth [19]. Not only does affirmation from the general parent community positively impact TGD children directly, it also makes it easier for the parents of TGD children to adopt gender-affirming attitudes toward their children, when these prevail in the broader parent community [20,21]. Importantly, a parent’s journey toward acceptance and support of their TGD child begins with them as a member of the general parent community [22]. Only once they have truly accepted the TGD identity of their child will they identify as “a parent of a TGD child.” So, efforts to promote acceptance and reduce transphobia in the general parent population supports TGD youth in two ways: (1) a more accepting general parent population will model acceptance for the parents of TGD youth, and (2) a more accepting general parent population results in gender-affirming interactions with TGD youth in school or social settings, thereby creating a more accepting environment in which all children can thrive [19-22].

A significant body of research suggests that social contact-based interventions may be the most effective way to reduce stigma [23-26]. Grounded in intergroup contact theory, this research suggests that exposure to the stories and experiences of transgender individuals can effectively reduce transphobia toward them [27-31]. A known stumbling block of this approach is the selection bias that limits exposure to the stories of TGD youth, since prejudiced individuals generally avoid intergroup contact [30]. Disseminating the stories of TGD youth to the general public, on the platforms where they readily consume information, still presents a major challenge.

In the postpandemic era, social media has become a primary source of health information for the public [32]. A 2021 scoping review in the *Lancet Digital Health* described social media as a “crucial communication tool” for public health [33]. Social media has proven effective for reaching broader and more varied audiences, including hard-to-reach populations [32]. As such, social media presents a powerful, emerging pathway for scaling the stories of TGD youth to the public.

Short, animated storytelling videos, disseminated via social media, emerged as a powerful digital approach to engaging diverse audiences around the world, during the COVID-19 pandemic [34]. Harnessing the appeal of human stories, enhanced with compelling soundtracks, this novel digital approach is easily shareable and thus readily scalable via social media [34,35].

This study investigated the effect of a short, animated storytelling video on transphobia in a sample of US parents. Our hypothesis was that exposure to the authentic narratives of TGD youth, animated to protect their identities, could effectively reduce transphobia and improve attitudes toward gender diverse children, thereby boosting affirmation and support for TGD youth, among parents in the general public.

## Methods

### Participants and Recruitment Procedure

From January to February 2024, we enrolled parents from the general US population using Prolific Academic, a crowdsourcing platform frequently used to recruit participants in online psychology and medical research [36]. Prolific supports high-quality data collection by ensuring consistency in demographic responses over time, screening for bots, blocking participants who hide their location, and assigning anonymous unique participant IDs [37,38]. For this trial, we recruited English-speaking parents, aged 18‐50 years, with at least one child, who were residing in the United States. Participants were recruited through the Prolific Academic platform. Before the initiation of this study, all participants reviewed a detailed information sheet, outlining the rationale for this study as well as all study procedures, and potential risks and benefits of taking part in this study. Those who agreed to participate were directed to complete this study’s procedures on Qualtrics, a secure online data-collection platform.

### Ethical Considerations

This study, which involved human participants, was performed in accordance with the ethical standards laid down in the 1964 Declaration of Helsinki and was approved by the Stanford University internal review board (IRB-#72761) on January 21, 2024. We followed the CONSORT (Consolidated Standards of Reporting Trials) guidelines [39], and this study was registered (#159248) with AsPredicted, a clinical trial registry created in 2015 by the Wharton School at the University of Pennsylvania [40].

Informed consent was obtained from all participants prior to enrollment in this study. Participants were provided with an information sheet that contained a detailed description of this study’s purpose, procedures, potential risks, and benefits. The information sheet was reviewed and approved by the Stanford IRB. Participants were informed that participation was voluntary and that they could withdraw at any time without penalty. The consent process was facilitated by Prolific Academic, the academic recruitment platform through which participants were recruited. Participants were paid US $2.40 for participation in this short study, at 2 time points and in accordance with the payment recommendations of Prolific Academic. Participants were assured that their responses would remain confidential and that any identifying data would not be shared with the research team by Prolific. The informed consent process was reviewed and approved by the Stanford IRB (#72761) ensuring compliance with ethical research standards.

### Randomization and Study Design

After consenting to take part in this study, participants were randomly assigned to one of two groups: short, animated storytelling video intervention group or a control group that received a simple fact sheet about TGD youth. We used a baseline survey to collect demographic information and we administered validated questionnaires to assess transphobia and attitudes toward TGD youth. We conducted the first round of surveys immediately post exposure to the short, animated storytelling intervention. Follow-up assessments, using the same surveys, were conducted 30 days later. To ensure accuracy and validity of the results, we included attention checks (eg, “For this question, please select the answer ‘Neither agree nor disagree.’”) and excluded participants who failed these. We also used a timer to confirm that participants in the control group read the TGD fact sheet and that those in the intervention group viewed the short, animated storytelling video before proceeding to the next screen. Each participant received US $2.40 for completing this study.

### Intervention

**Figure 1.**
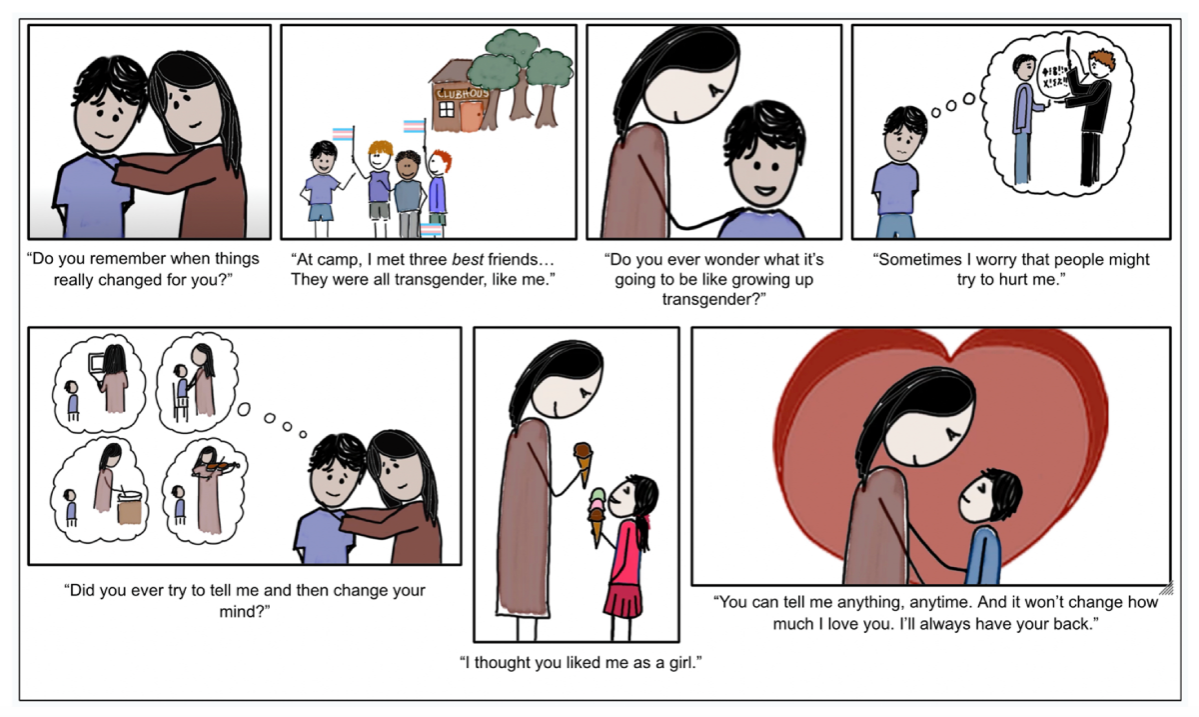
Selected scenes and quotations from the short, animated storytelling intervention video shown to reduce transphobia in US parents in this online, randomized controlled trial.

Our novel, digital intervention used a short, animated storytelling video to capture an authentic prerecorded conversation between a mother and her transgender child. We chose this video because it follows the principles of social contact-based interventions, which foster identification and emotional engagement. A significant body of research suggests that these principles have proven effective in reducing stigma and transphobia by exposing viewers to the real-life stories and experiences of marginalized individuals [23-30,41]. In the conversation featured in our intervention, a mother asks her son to recall experiences related to his gender identity, including his early hesitations to confide in her that he was transgender. The child then asks his mother about her worries, and she shares these candidly. The identities of the speakers are concealed at all times, and we received written permission to use the audio recording from the speakers. The scenes and characters used to represent the speakers were drawn by hand on a Wacom tablet, by our coauthor (MA), and edited by the Educational Technology Group at Stanford Medicine. Figure 1 shows selected scenes and quotations from the intervention video.

The short, animated video tested in this trial can be previewed on YouTube [42].

### Instruments

The primary outcome was transphobia toward TGD youth, which was measured using the total score of the Transgender Stigma Scale (TSS) developed by Madera et al [43]. We adjusted the wording of the scale items to assess attitudes toward transgender children (eg, “Boys who act like girls should be ashamed of themselves,” and “Children should play with toys appropriate to their own sex”). The TSS is scored along a 5-point Likert scale ranging from “strongly disagree” (1) to “strongly agree” (5). Higher scores indicate greater transphobia. The TSS is highly reliable and has a Cronbach α of 0.89 [43].

As secondary outcome, we measured attitudes toward TGN children, using the “gender thermometer,” a tool we previously developed to assess attitudes regarding sexual orientation and gender diversity [27]. The thermometer provides the following prompt: “Using a scale from zero to 100, please tell us about your personal feelings toward transgender boys or girls. As you do this task, think of an imaginary thermometer. The warmer or more favorable you feel toward the group, the higher the number you should give it. The colder or less favorable you feel, the lower the number. If you feel neither warm nor cold toward the group, rate it 50.” We asked respondents about their attitudes toward (1) boys or girls and (2) transgender boys or girls. Higher ratings indicate more positive attitudes toward the group and lower ratings indicate less positive attitudes. Researchers in the fields of political science and psychology have frequently used feeling thermometers to assess attitudes toward diverse groups, including sexual minorities [44].

### Analysis

The main outcome measures were changes in total TSS scores and gender thermometer scores, from baseline to post intervention and from baseline to 30-day follow-up. We calculated our sample size based on our previous studies [27,45-48]. We used Pearson chi-square and one-way ANOVA tests to compare sociodemographic characteristics between the short, animated storytelling video intervention and control groups. We used repeated-measures ANOVA tests to compare the mean transphobia score between the groups at 3 time points. Next, we used a one-way ANOVA test to compare changes from baseline scores, at post intervention, and at 30-day follow-up. We used independent *t* tests (2-tailed) to compare gender differences at baseline. Data were analyzed using SPSS (version 29.0; IBM Corp) [49].

## Results

### Sample Characteristics

**Figure 2.**
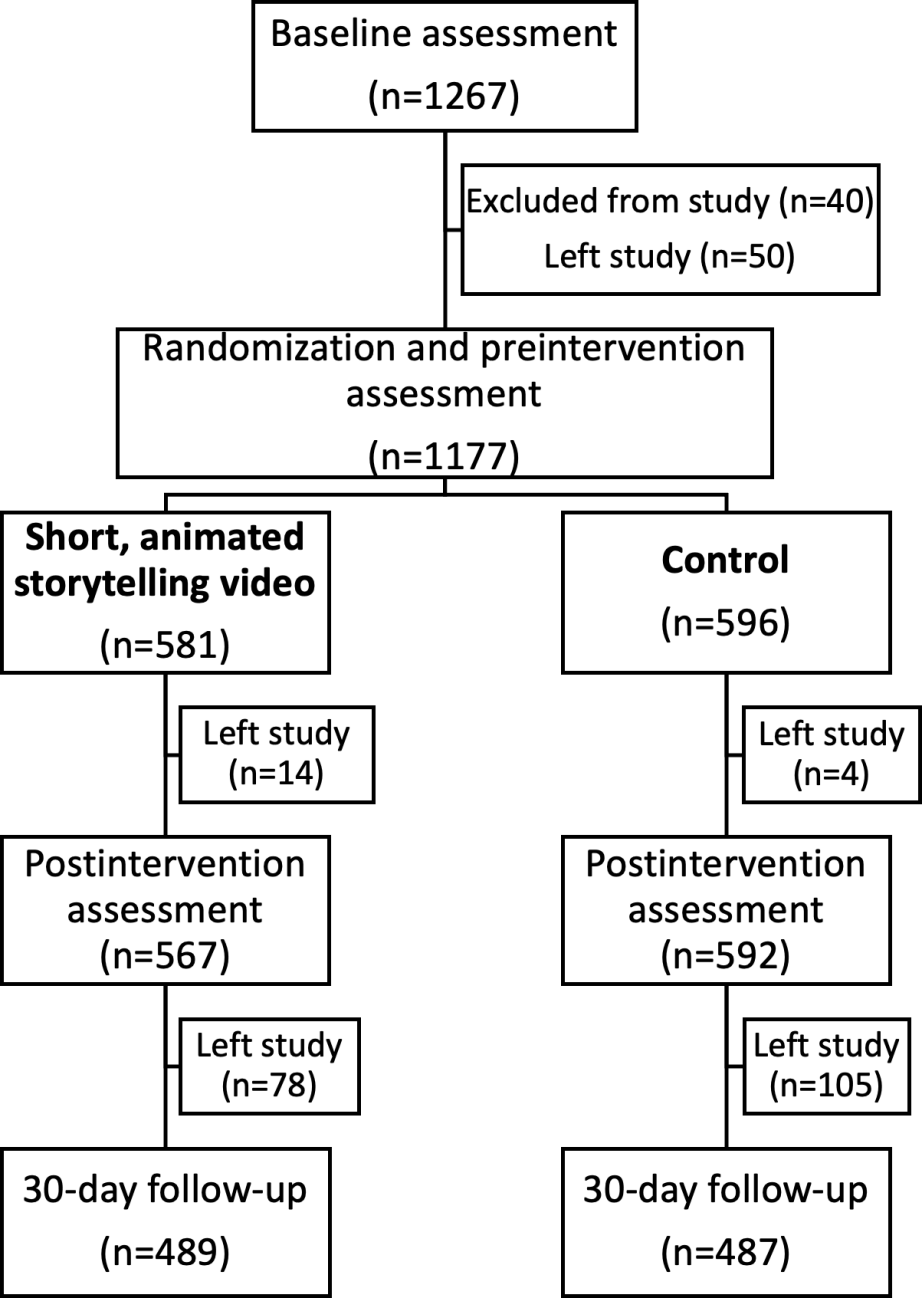
Study design and flow for our online, randomized controlled trial testing the effect of a short, animated storytelling video intervention on transphobia in US adults.

We recruited 1267 US parents, through the Prolific Academic online research platform. In total, 1177 parents completed the preintervention assessment after we excluded 40 (3%) who failed attention tests and 50 (4%) who left this study. Of these, 1159 (98%) completed the postintervention assessment and 976 (83%) completed the 30-day follow-up assessment. Figure 2 shows the design and flow of this study.

Sociodemographic characteristics and completion rates did not differ significantly between groups (Table 1), nor did baseline characteristics differ between follow-up completers and noncompleters. Mean participant age was 39 (SD 6.9; range 20‐50) years. More than half of participants self-identified as female (n=653, 56%), 510 (43%) male, and 14 (1%) transgender or nonbinary. Overall, 110 (9%) participants self-identified as Hispanic, 192 (16%) non-Hispanic Black, 789 (67%) non-Hispanic White, 57 (5%) non-Hispanic Asian, 8 (1%) non-Hispanic Native American, and 18 (2%) other (Table 1).

**Table 1.** Demographic characteristics of study participants.

Items		Brief animation (n=581)	Narrative (n=596)	Total (N=1177)	Statistic	
					Pearson *χ*^2^ (*df*)	*P* value
Age (years), mean (SD)		39.1 (7.1)	39 (6.8)	39 (6.9)	0.18 (*1175*)^a^	.86
**Gender, n (%)**					2.62(*2*)	.27
	Female	336 (58)	317 (53)	653 (56)		
	Male	238 (41)	272 (46)	510 (43)		
	Transgender or nonbinary	7 (1)	7 (1)	14 (1)		
	Prefer not to answer	0 (0)	0 (0)	0 (0)		
**Race and ethnicity, n (%)**					9.35 (*6*)	.16
	Hispanic	62 (11)	48 (8)	110 (9)		
	Non-Hispanic Asian	32 (6)	25 (4)	57 (5)		
	Non-Hispanic Black	97 (17)	95 (16)	192 (16)		
	Non-Hispanic Native American	3 (1)	5 (1)	8 (1)		
	Non-Hispanic White	378 (65)	411 (69)	789 (66)		
	Non-Hispanic other^b^	6 (1)	12 (2)	18 (2)		
	Prefer not to answer	3 (1)	0 (0)	3 (0)		

a Independent *t* tests.

b Non-Hispanic other: multiracial (n=15), Native Hawaiian (n=1), Jewish (n=1), and South Asian (n=1).

### Intervention Effects

**Figure 3.**
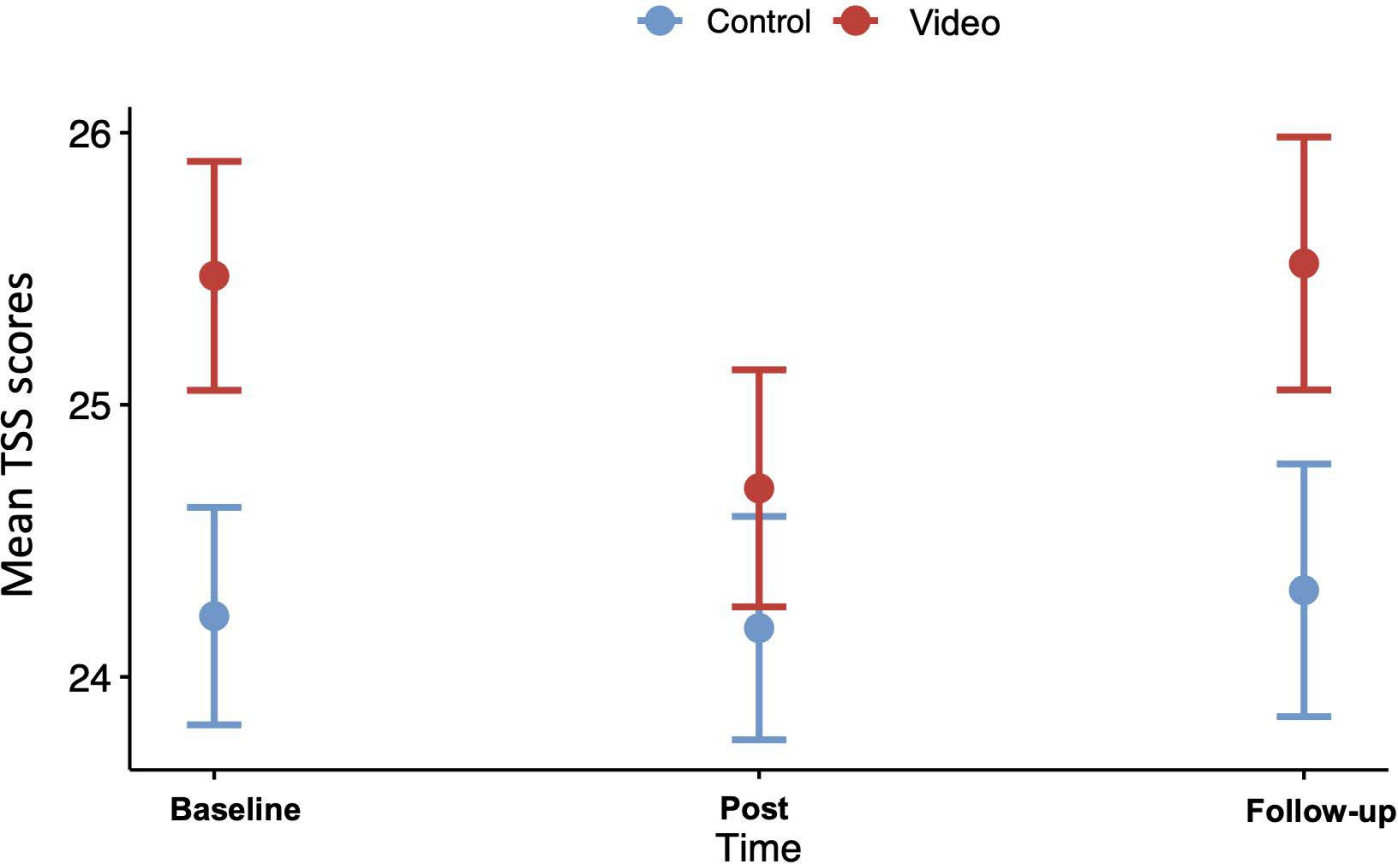
Mean scores on the TSS between short, animated storytelling video intervention (n=588) and control (n=598) groups at 3 time points in our online RCT measuring the effect of a short, animated storytelling intervention on transphobia in US adults. Follow-up surveys completed after 30 days; higher scores indicate greater transphobia; TSS (range 10‐50). Repeated measure ANOVA (*P*=.02). RCT: randomized controlled trial; TSS: Transgender Stigma Scale.

We observed significant differences in outcomes between this study’s groups. Figure 3 presents mean TSS scores of this study arms at 3 time points, showing that the control arm changed minimally across time points, in contrast to the short, animated storytelling video intervention arm, which showed a strongly significant decrease in transphobia immediately post intervention. This effect was no longer evident at 30 days post intervention. A 2 × 3 group-by-time ANOVA showed a significant group-by-time interaction in mean TSS scores (*F*_2,1_=3.7, *P*=.02). One-way ANOVAs revealed significant between-group changes between the short, animated storytelling video and control groups from baseline to post intervention (*F*_1_=27.4, *P*<.001), but not from baseline to the 30-day follow-up.

**Figure 4.**
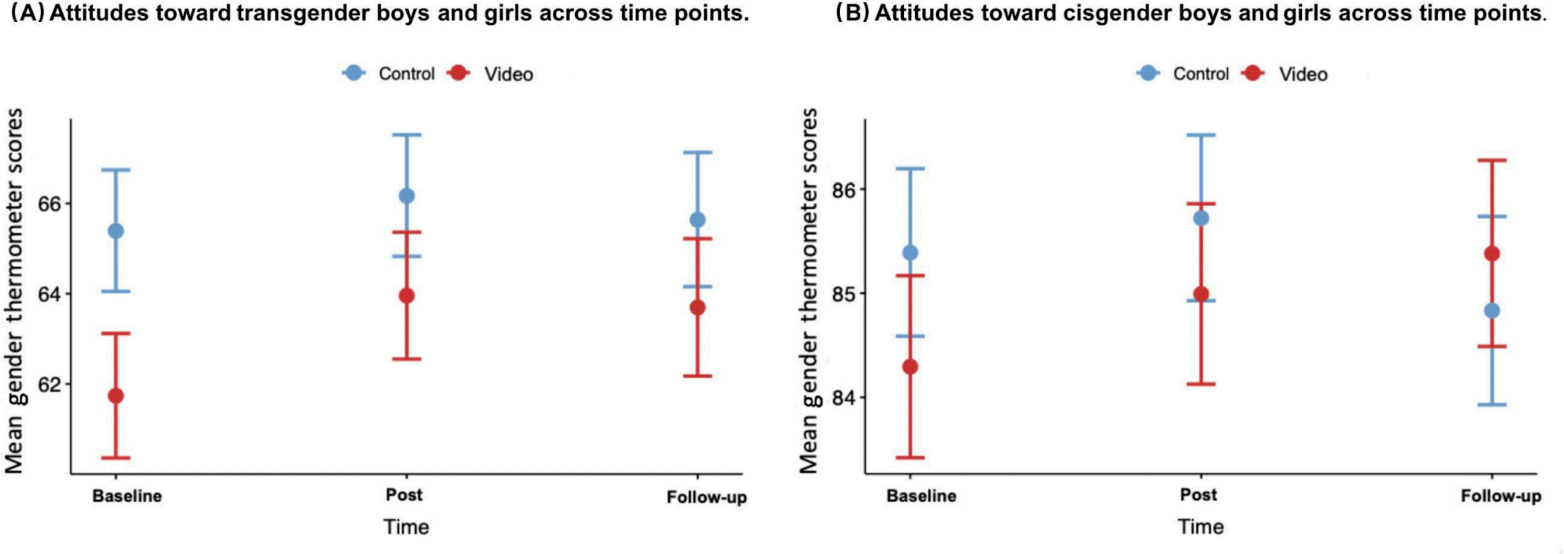
Gender thermometer scores toward (A) transgender children and (B) cisgender children, at 3 time points in our online RCT measuring the effect of a short, animated storytelling intervention on transphobia in US adults. Follow-up surveys completed after 30 days; higher scores indicate more favorable attitudes; gender thermometer (range 0‐100). RCT: randomized controlled trial.

Figure 4 presents mean gender thermometer scores, by study arm, over time. Figure 4A shows changing attitudes toward transgender boys or girls. Attitudes of participants in the short, animated storytelling video intervention arm improved significantly over time and this improvement was sustained at the 30-day time point. In contrast, we observed a slight improvement in the control arm, which was not significant, and showed a subsequent rebound at the 30-day follow-up.

We did not find a 2 × 3 group-by-time interaction. However, independent *t* tests showed a significant difference between the changes from baseline to post intervention between short, animated storytelling video intervention and control groups (change of 2.2, SD 9.7 vs 0.8, SD 6.5; *t*=3, *t*_df_=1151 *P*=.003). We did not observe an effect of the intervention on attitudes toward cisgender boys or girls (Figure 4B).

### Participant Gender Differences

**Figure 5.**
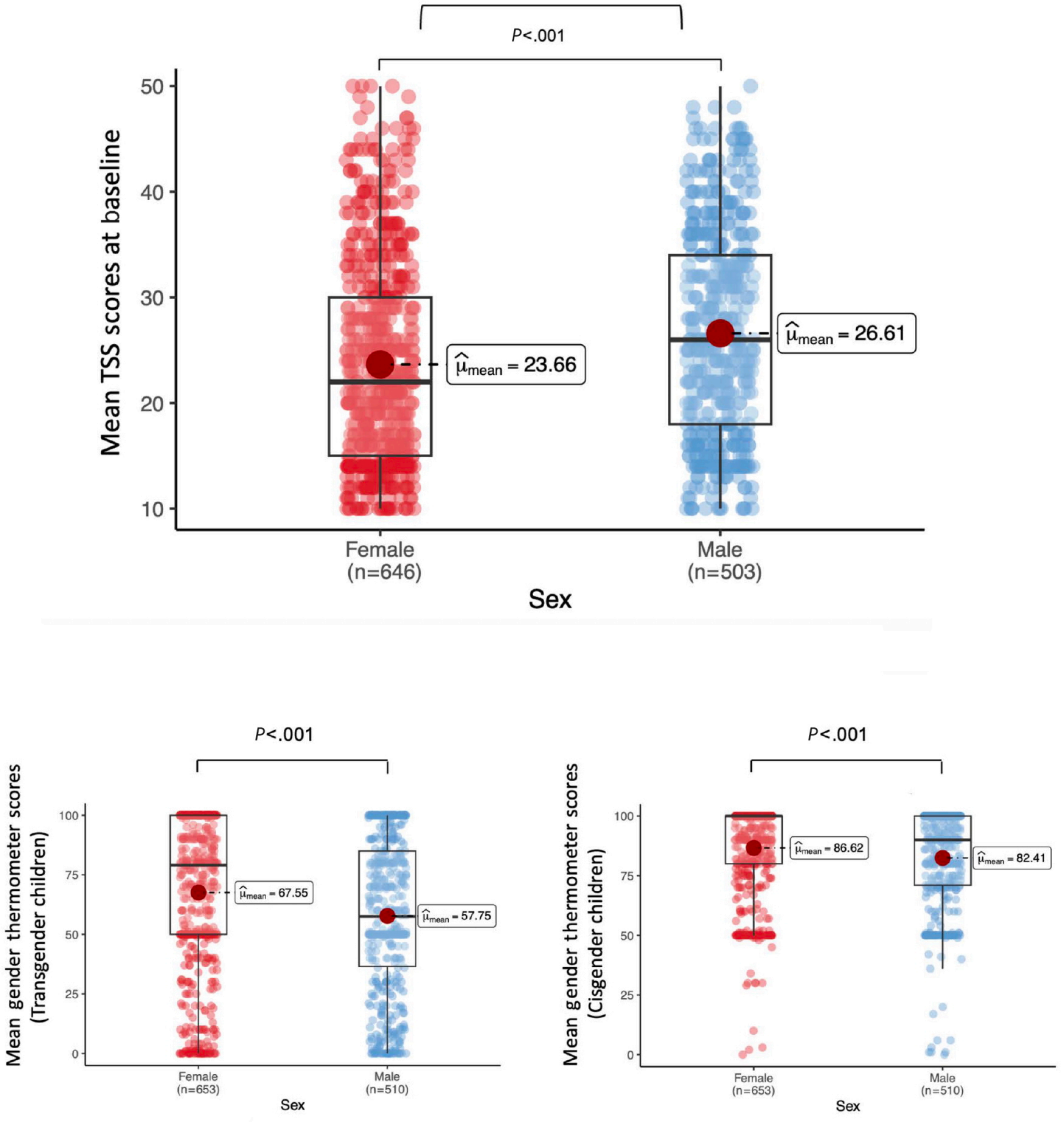
Gender differences (TSS and gender thermometer) at baseline in our online RCT measuring the effect of a short, animated storytelling intervention on transphobia in US adults. Higher scores indicate greater transphobia; TSS (range 10‐50). Higher scores indicate more positive attitudes; GT (range 0‐100). GT: gender thermometer; RCT: randomized controlled trial; TSS: Transgender Stigma Scale.

Figure 5 presents baseline differences between men and women on the TSS (26.6, SD 9.7 vs 23.7, SD 9.8; independent *t* tests*: t*=5.1, *t*df=1161, *P*<.001) and on the gender thermometer both for transgender boys or girls (57.7, SD 32 vs 67.5, SD 33.1, *t*=5.1, *t*df=1161, *P*<.001) and cisgender boys or girls (82.4.7, SD 21 vs 86.6, SD 19.7, *t*=3.5, *t*df=1161 *P*<.001). The observed baseline differences between men and women remained constant across time points in both study groups.

## Discussion

In this study, we conducted a large-scale, randomized, controlled experiment to document the potential of a novel, digital approach—short, animated storytelling—to reduce transphobia and improve attitudes toward TGD youth among US parents. Within our study population, we observed significantly reduced transphobia and significantly improved attitudes toward TGD youth, immediately after viewing the 2.5-minute short, animated storytelling video intervention. The effect of this short video was no longer evident one month after the intervention, suggesting that repeated, short exposures to similar short, animated storytelling video content may be necessary to sustain transphobia reductions and improvements in attitudes over time. One potential approach is to develop and disseminate a series of short, animated storytelling videos featuring the authentic lived experiences of several TGD young people. This scalable, digital approach could be implemented through a smartphone-based ecological momentary intervention. Evaluating the effect of such a series could be a meaningful future research direction in the fields of both digital health communication and transgender health.

During the COVID-19 pandemic, our coinvestigator (MA) developed the short, animated storytelling approach, a novel digital modality for scaling health information worldwide. Early short, animated storytelling COVID prevention videos reached more than 15 million people via social media within 4 months [35]. High voluntary public engagement with short, animated storytelling content was also documented in randomized, controlled trials [50,51]. As short, animated storytelling videos have proven effective for engaging diverse adult audiences, they present a potentially promising new method for engaging the public in the stories of TGD youth. The findings of this study support the potential for using short, animated storytelling to reduce transphobia and elicit empathy. Animation also protects the identities of the TGD storytellers—an important consideration, since many of these young people still live in “stealth” [52].

Intergroup contact theory, and recent research grounded on this theory, support the stigma-reducing potential of social contact with TGD individuals [27,28,30,31,53,54]. To date, interventions designed to reduce transphobia have been difficult to scale broadly to the public because (1) they involve face-to-face or online synchronous interactions, (2) they involve a significant time commitment and some form of active “opting in,” thereby self-selecting for people who already have low stigma, or (3) they lack the production value needed to make them highly engaging—one of the keys to scalability [55]. The short, animated storytelling video tested in this study packages a theory-driven approach to stigma reduction (the sharing of human stories) in a readily scalable form of health message (short, animated videos that can be shared via social media) to overcome a critical health challenge (lack of support and affirmation for TGD youth). Anecdotally, and supporting the potential for easy scalability, one participant in our study sent a message to the research team:

As the father of a child with 2 trans friends, this video is amazing and I showed it to my daughter, who is 8 and she completely loved it. She asked if we could send it to her friends’ parents, who called us and thanked us. So I wanted to let you know this was helpful and well thought. I can’t thank you enough!

In this study, we also observed significant differences in baseline transphobia between men and women. Aligned with prior research, men in our study scored significantly higher on transphobia than did women, and these differences persisted across time points [11,56]. Other researchers have proposed that threatening gender norms can trigger a perceived loss of social status among men more than women [57]. Men may also be more likely to feel that transgender individuals pose a threat to their own gender identity and masculinity [58]. Future interventions could include story-based content that is tailored toward men. Featuring the stories of fathers, who are supporting of and affirming to their transgender children, may be particularly important. We also note that, to date, much of the research on family support of TGD youth focuses on parents, but there is an emerging body of research on the attitudes of other family members (eg, grandparents and siblings) toward transgender youth. If parents are not supportive, these other family members may be able to play a vital role in protecting transgender youth and the approach described here could also work with a wider range of target audiences.

A limitation of this study is the fact that our sample was recruited via an online research platform, suggesting that the sample may not be entirely representative of the general population in the United States. However, considering the intended, digital dissemination route for our intervention (social media), we feel this population is similar enough to our target population to allow us to gain some meaningful insights and draw relevant conclusions.

Our findings highlight the important role of authentic storytelling, packaged in scalable, accessible digital formats, for reducing transphobia toward TGN youth. These results also add to the growing field of research on short, animated storytelling, an innovative digital approach to rapidly scaling health information worldwide. Finally, these findings underscore the potential for light-touch, scalable video content to improve attitudes toward TGN youth within our society. Simply put, adults can be critical determinants of better health outcomes for TGD youth, simply by affirming their right to live and express themselves. Using authentic storytelling to engage and elicit empathy, and social media to broadly reach adults across demographics, we have the potential to catalyze a shift toward affirmation and support within society—a shift that could help children of all gender identities to survive and thrive.

## Supplementary material

10.2196/66496 Checklist 1 CONSORT-EHEALTH checklist (V 1.6.1).

## Abbreviations

CONSORT: Consolidated Standards of Reporting Trials
IRB: internal review board
LGBTQ: lesbian, gay, bisexual, transgender, queer
TGD: transgender and gender diverse
TSS: Transgender Stigma Scale
